# Supplementary material for: Outcome of burn injury and its associated factors among burn patients attending public hospitals in North Showa Zone, Ethiopia: A cross-sectional study
Source: PLOS Glob Public Health. 2024 Sep 16;4(9):e0003682. doi: 10.1371/journal.pgph.0003682 (PMC11404801; doi:10.1371/journal.pgph.0003682)
Supplement: S1 Text — (DOCX) [file pgph.0003682.s001.docx]

**Annex- English version questionary for outcome of burn injury and its Associated Factors among Burn Patients Attending Public Hospitals in North Showa Zone, Ethiopia, 2023**

**Part I- Socio-demographic factors**

1. Identification number___________

2. Medical Record Number___________

3. Date attended at public hospital for health care (DD/MM/YYYY in E.C) ____________in E.C

4. Address of patient? Region/City __________________ Zone/Sub city ___________­­­­­­___ District/Tow____________________

5. Age of patients (in complete years) ________________ years

6. Sex 1.Male 2. Female

7. What is main occupation of the patient? _________________

8. Marital Status

1. Single

2. Married

3. Divorced

4. Widowed

5. Other/specify _________________________

**Part II. Clinical Factors**

9. Duration of burn injury prior to hospitalization (in hours)? ________________hours

10. Place where a patient was found when burn occurred/happened to him/her?

1. Home

2. Street

3. School

4. Work place

5. Others (specify) ____________________

11. What was the main cause of burn injury?

1. Flame

2. Scalds

3. Steam

4. Chemical

5. Electrical

6. Others (Specify) __________________

12. If thermal burn, which type of thermal burn is it?

1. Stove explosion

2. Fell in fire

3. Hot water

4. Cloth caught fire

5. Others (Specify) __________________

13. If electrical burn, which type of electrical burn is it?

1. Low voltage

2. High voltage

3. Flash burn

4. Other/specify) ___________________

14. If chemical burns, what type of chemical burn is it?

1. Acid

2. Alkali

3. Others (Specify) _________________

15. What was main circumstance surrounding the burn:

1. Accident

2. Assault

3. Suicidal

4. Others (Specify) _______________

16. How was the depth of burn injury?

1. Superficial/first degree burn

2. Partial thickness/second degree burn

3. Full thickness/third degree burn

17. Extent of burn injury (% of total burn surface area burned): _________

18. Body parts affected by burn injury (multiple response are possible)

1. Head and neck

2. Upper extremities

3. Lower extremities

4. Perineum

5. Anterior trunk

6. Posterior trunk

7. Other/specify

19: Was the burn injury is inhalation burn? 1. Yes 2. No

20. Presence of comorbidity? 1. Yes 2. No

21. If yes for comorbidity, what is/are comorbidities (multiple response are possible)?

1. Epilepsy

2. HIV/AIDS

3. Diabetes

4. CVS disease

5. Alcohol

6. Pregnancy

7. Others (specify) ________________

**Part III. Healthcare related factors**

22. Does fluid and electrolyte replacement given to burn patients upon admission? 1. Yes 2. No

23. Pre hospital intervention provided (first aid) to burn patients? 1. Yes 2. No

24. If yes what care was provided before hospitalization?__________________

25. Length of stay with injury prior to hospitalization (in day’s) ______

26. Length of hospital stays in day’s from admission to discharge? ______

27. What intervention (s) provided to burn patient during hospitalization (multiple response are possible)?

1. Fluid replacement

2. Burn wound management

3. Antibiotic

4. Pain management

5. Tetanus toxoid

6. Surgery

7. Other (specify) __________________

28. If the surgery intervention, what type of surgery was performed?

1. Fascioctomy

2. Escharotomy

3. Skin graft

4. Skin flap

5. Contracture release

6. Debridement

7. Other/specify___________________

29. Outcome of a patient who had sustained a burn injury?

1. Outcome of burn injury discharged with complication

2. Outcome of burn injury discharged without complication

30. If the outcome was recovery with complication, what was the complication?

1. Scarring or disfigurement

2. Disability

3. Others/specify _____________
