# Supplementary material for: Outcome of burn injury and its associated factors among burn patients attending public hospitals in North Showa Zone, Ethiopia: A cross-sectional study
Source: PLOS Glob Public Health. 2024 Sep 16;4(9):e0003682. doi: 10.1371/journal.pgph.0003682 (PMC11404801; doi:10.1371/journal.pgph.0003682)
Supplement: Attachment — Submitted filename: PGPH-D-23-01362_reviewer.pdf [file pgph.0003682.s004.pdf]

PLOS Global Public Health

Outcome of Burn Injury and its Associated Factors among Burn Patients Attending at Public Hospitals in North Showa Zone, Ethiopia: A Cross-Sectional Study

--Manuscript Draft--

**Manuscript Number:**	PGPH-D-23-01362
**Article Type:**	Research Article
**Full Title:**	Outcome of Burn Injury and its Associated Factors among Burn Patients Attending at Public Hospitals in North Showa Zone, Ethiopia: A Cross-Sectional Study
**Short Title:**	Outcome of Burn Injury and its Associated Factors
**Corresponding Author:**	Abebe Nigussie Ayele, MSc<br>Debre Berhan University<br>Db, ETHIOPIA
**Order of Authors:**	Ejigu Mulugeta Shewaye, MPH
	Akine Eshetie Abosetugn, Assistance professor
	Mekasha Getnet, Msc
	Abebe Minda
	Abebe Nigussie Ayele, Msc
	Mitiku Tefera, Msc
**Keywords:**	Burn injury; burn patients; outcome; Ethiopia
**Abstract:**	Background: Burn injury is a major contributor to morbidity and mortality in Sub-Saharan Africa including Ethiopia, require regular interventions. There is limited evidence on outcome burn injury status in Ethiopia including the study area.<br>Objective: To assess the outcome of burn injury and its associated factors among burn patients attending public Hospital in North Showa zone, Ethiopia.2023<br>Method: An institution-based cross-sectional study was conducted among 420 burn patients in public Hospital in North Showa zone, from April to May, 2023. Systematic random sampling was used to select patients' cards. Structured checklists were used to extract data from burn patients' medical records. Data were entered using Epi-data version 4.6 and analyzed using SPSS version 25 variables with a p- value of ≤ 0.25 in the bivariable logistic regression were included in the multivariable logistic regression. A p- value of ≤ 0.05 within 95% confidence interval in the multivariable logistic regression was used to declare significant association between the dependent variable and the independent variables. Adjusted odd ratio was used to show the strength of association between the dependent and independent variables.<br>Result: In this study 40.9% (95% CI: 36.5– 45.6) were discharged with complication. Patients who had pre-hospital intervention was nearly four times (AOR= 3.8, 95% CI, 1.11-13.25) and Patients burn with scalds was four (AOR=4.3, 95% CI, 1.52-12.32) times more likely to develop discharged with complication. Patients who had provided fluid and electrolyte were 76% and the occurrence of total body surface area burn <20% were 66% less likely to develop the outcome burn injury discharged with complication.<br>Conclusion: This study demonstrates a significantly higher level of the outcome of burn injury discharged with complication, leading to death and other worst outcomes. Having pre-hospital intervention, patients with scalds burn, Early fluid and electrolyte provided and having total body surface area <20% were significant factors associated with poor outcome of burn injury. Therefore health education on pre-hospital intervention and the cause of burn and providing fluid and electrolyte for patients total body surface area affected more than twenty percent.
**Additional Information:**	
**Question**	**Response**
**Financial Disclosure**	The authors received no specific funding for this work.

Powered by Editorial Manager® and ProduXion Manager® from Aries Systems Corporation

Enter a financial disclosure statement that describes the sources of funding for the work included in this submission and the role the funder(s) played. This includes grants and any commercial funding of the work or authors.<br><br>This statement will be typeset if the manuscript is accepted for publication.<br><br>*Please review the submission guidelines and the instructions link below for detailed requirements and guidance.*	
**Competing Interests**<br><br>On behalf of all authors, disclose any competing interests that could be perceived to bias this work.<br><br>This statement will be typeset if the manuscript is accepted for publication.<br><br>*Please review the instructions link below and PLOS Global Public Health's competing interests policy to determine what information must be disclosed at submission.*	The authors declare that they have no competing interests exist
**Data Availability**<br><br>Before publication, Authors are required to make fully available and without restriction all data underlying their findings. Please see our PLOS Data Policy page for detailed information on this policy.<br><br>A **Data Availability Statement**, detailing where the data can be accessed, is required at first submission. Insert your Data Availability Statement in the box below.	We will submit data set via your address when you ask because of no data restriction for our finding conclusion

Please see the data reporting section of our submission guidelines for instructions on what you need to include in your Data Availability Statement.<br><br>This statement will be typeset if the manuscript is accepted for publication.<br><br>*PLOS allows rare exemptions to address legal and ethical concerns. If you have legal or ethical restrictions, please use the box below to detail these in full sentences for the Journal team to consider.*	

Powered by Editorial Manager® and ProduXion Manager® from Aries Systems Corporation

Manuscript Click here to access/download;Manuscript;Manscript.docx

Outcome of B[illegible]rn Injury and its Associated Factors among Burn Patients Attending at Public Hospitals in North Showa Zone, Ethiopia: A Cross-Sectional Study

Ejigu Mulugeta Shewaye[1], Akine Eshete Abosetugn[2], Mekasha Getnet[3], Dr. Abebe Minda [4], Abebe Nigussie Ayele [5],Mitiku tefera[6]

Address

Email; ejigu.mulugeta1234@gmail.com

[1]Departments of Nursing, Debre Berhan University, Ethiopia,

Email; _akine.eshete@yahoo.com

[2] Department of Nursing, Debre Berhan University, Asrate Woldeyes Health Science Campus, Ethiopia

Email; mekashagetnet@gmail.com

[3]Department of Nursing, Debre Berhan University, Asrate Woldeyes Health Science Campus, Ethiopia

Email: abebe2014nigussie@gmail.com

[4]Department of Nursing, Debre Berhan University, Asrate Woldeyes Health Science Campus, Ethiopia

Email: abe.minda@yahoo.com

[5]Department of Nursing, Debre Berhan University, Asrate Woldeyes Health Science Campus, Ethiopia

mitikutefera2632@gmail.com

[6] Department of Midwifery Debre berhan health science college, Amhara Ethiopia.

Abstract

Background: Burn injury is a major contributor to morbidity and mortality in Sub-Saharan Africa, including Ethiopia, and requires regular interventions. There is limited evidence on the outcome of burn injury status in Ethiopia, including the study area.

Objectiv[illegible] assess the outcome of burn injury and its asso[illegible]d factors among burn patients attending public Hospital in North Showa zone, Ethiopia.2023

Method[illegible] institution-based cross-sectional study was conducted among 420 burn patients in a public Hospital in the North Showa zone from April to May 2023. Systematic random sampling was used to select patients' cards. Structured checklists were used to extract data from burn patients' medical records. Data were entered using Epi-data version 4.6 and analyzed using SPSS version 25. Variables with a p-value of ≤ 0.25 in the bivariable logistic regression were included in the multivariable logistic regression. A p-value of ≤ 0.05 within the 95% confidence interval in the multivariable logistic regression was used to declare a significant association between the

dependent variable and the independent variables. An adjusted odd ratio was used to show the strength of association betwe[illegible] the dependent and independent variables.

Result: In this study, 40.9% ([illegible]% CI: 36.5–45.6) were discharged with complications. Patients who had pre-hospital intervention were nearly four times (AOR = 3.8, 95% CI, 1.11–13.25) more likely to develop complications, and Patients who burned with scalds were four (AOR = 4.3, 95% CI, 1.52-12.32) times more likely to be discharged with complications. Patients who [illegible]ived fluid and electrolytes were 76% less likely to develop the outcome [illegible] injury discharged with complication, and patients who received total body surface area burn 20% less likely.

Conclusion: This study demonstrates a significantly higher leve[illegible] outcome for burn injuries discharged with complications, leading to death and other worst outcomes. Therefore, health education on pre-hospital intervention, the cause of burns, and providing fluid and electrolytes for patients total body surface area affected more than twenty percen[illegible]

Keywords: Burn injury, burn patients, outcome, Ethiopia.

Introduction

Burn is a term [illegible] to describe skin and tissue damage brought on by a fire, scald, electrical shock, chemical, or radioactive radiation. Due to the prolonged hospitalization for rehabilitation and the treatment of wounds and scars, burns are the most expensive traumatic injuries[1]. Large burn victims have many of the same physiological reactions as victims of major trauma, particularly venous thromboembolism [2]. It is one of the frequent and unpleasant forms of trauma in addition to being a substantial worldwide health problem that increases morbidity and death and causes significant material, psychological, and financial damage [3]. Burns are damaging wounds that are linked to morbidity, a reduction in quality of life, and emotional well-being [4]. Mortality and morbidity from burns remain high, particularly in develop[illegible] countries [5]. These areas are also areas where the lack of socio-economic growth and geopolitical instability create additional barriers not only to the delivery of health care, but also to the acquisition of continuous professional development [6]. Burn[illegible] estimated to be the root cause of 180,000 deaths worldwide, most of which currently occur in low- and middle-income countries [7]. Burns are the fourth leading cause of trauma in the world and are common in developing countries [8]. Burn injury incidence and mortal[illegible]ffect all regions of the world, but are most concentrated in middle- and low-income countries [9]. Burns are a major public [illegible]h problem worldwide [10]. The fatality rate for child burn victims in central Malawi is 27% [11].

The study shows that the magnitude of burns with complications in [illegible]opia was 14.9% , the highest risk groups compared to children age groups and the elderly [12]. In almost all age groups, the prevalence of burns is higher in women [13]. The in[illegible]se in burn risk in 3rd degree burns was more likely in case of wound infection and the risk of [illegible]ection was also considered in burn surgery [14]. Wound i[illegible]ion and septicemia were the two most common complications in burn patients [15]. A burn contains both nociceptive and neuropathic pain [illegible]ponents, so managing a burn [illegible]ne of the most difficult challen[illegible] for the firefighting team[16]. Treating a burn requires nursing staff many hours of wound care, possi[illegible]multiple surgical procedures, and expensive hospitalization [17]. Burns are the most painful and require special attention and are considered a major public health problem [18]. Burns are the fourth most common type of trauma causing death and disability after traffic accidents, falls and interpersonal violence [19]. Globally, burns are one of the leading causes of disability, accounting for more than 8 million disability-adjusted life years (DALYs) [20]. In the United States, the direct cost burden of treating children with burns has exceeded $211 million. In Norway, the expenses of giving hospital burns exceeded 10.5 million euros. In South Africa, an estimate[illegible]$26 million is spent annually on burn injury, but indirect costs such as lost wages, long-term treatment for disfigurement and emoti[illegible] trauma, and the contribution of family resources also contribute to socioeconomic impacts [illegible]. Currently, most burn centers around the world care for patients based on a protoco[illegible]]. In the 20th century, advances in burn care lead to an increased focus on reducing burn morbidity. [23]. Burns often result in significant psychological, educational and social stigma, and the resulting changes are exacerbated by many factors, including the circumstances of the burn, severity a[illegible]ocation of the injury, personality characteristics of the injured person[illegible] access to support interpersonal communication, and social relations [24]. Both the patient and their family may experience physical and emotional suffering as a result of the burns and treatment [25]. [illegible]ns are the second most common injury in ru[illegible]Nepal, accounting for 5 percent of trees [7]. It is estimated that over a million people burn in Africa each year and 18% of hospitalizations are due to burns [26]. Burns are a common source of harm in developing countries including Ethiopia [27]. The incidence of burns varies by country and population group, and the severity of the burn depends on the degree of heat, durati[illegible]f exposure, and thickness of the skin [28]. Burns occur mainly at hon[illegible]d in the workplace. As in Bangladesh and Ethiopia, show that 80-90% of burns occur in the home [29].In Ethiopia, the outcome of burn injury and associated factors among burn patients were not evidently described.

Hence, it needs more study to address the possible output of burn and sustaining uniform treatment protocol in health facil[illegible] Therefore, this study was assesses the outcome of burn injury and its associated factors at public hospital of North Shewa Zone, Amhara regional state.

Methods

Study design, setting and period

An institution-based cross-sectional study was conducted among 420 burn patients in public Hospi[illegible] North Showa zone, from April to May, 2023. Debre Berhan City is located 130-kilo meters from Addis Ababa, the capital city of Ethiopia and 696 km away from the Bahirdar city [illegible]ional state of Amhara. The total pop[illegible]on of North Sh[illegible] Zone was 2,322,148 of whom thos[illegible]71,510 are men and 1,150,638 women for the year 2019.According to Zonal Health Office Report, North Shewa has 164 private clinics, 97 governmental health centers, 391 health posts, 8 primary Hospitals (of which two [illegible] private primary hospitals), 2 general hospitals, and one comprehensive specialized hospital North Shewa Zone has a total of 10 public and 2 private hospitals.

Populations

Source Population: All patients who visited public hospital in North shoa zone

Study population: All burn patients who attended N[illegible]h shewa zone in selected public hospital from the 1st of January 2018 to December 30th 2022 in North Showa zone, Amhara, Ethiopia.

Inclusion and Exclusion criteria

Inclusion criteria

All burn patients who attended North shewa zone public hospital, registered on patient card/medical record folders in hospital and his/her card/record was available in medical record unit/card room were included.

Exclusion criteria

Burn patients with incomplete records/information on either the burn or miss diagnosis upon review were excluded from the study.

Sample Size Determination and Sampling Techniques

Sample size for this study was determined by single population formulas. A study of outcome of burn injury and associated factor among patient visited at Addis Ababa, public hospital, the

prevalence (p=54.7%) [12] ,with 5% margin of error, 95% CI and 10% non-response rate were considered. By adding a 10% non-response rate, the total sample size becomes **420**.

There are 10 public hospitals in the North Showa Zone; from these, five were selected using a simple random sampling method. The medical record numbers (MRN) of the burn patient were used as a sampling frame. The sampl[illegible]s allocated proportionally for each hospital. Study participants were selected consumin[illegible] a systematic random sampling technique. First, determined the sampling interval (K) value by dividing the total admitted burn patient in the study period by the total sample [illegible] which gives 2.17 ≈ 2 as shown in Figure 2.

Variables (seen in Figure 1)

Dependent variable

Outcome of Burn Injury discharge with complication or without complication

Independent variable

Socio demographic factors

Health factors

Clinical factors

Operational definitions

Discharge with complication: In this study the patients have been discharged with one of the following; like contracture, disfigurement, amputated, scar of skin graft and death as reported on the chart considered as poor outcome of burn injury.

Burn patients[illegible]atient who sustained burn injury.

Burn injury: Is injury of skin or other tissue caused by thermal, radiation, chemical or electrical contact [30].

Type of burn injury: are classified by depth (superficial and deep partial thickness, and full thickness and TBSA [illegible]nvolved.

Outcome of burn injury: when the patients after Hospital management was discharged with complication or without complication as reported on the chart [31].

Data collection procedures

Data were collected using structured checklist adopted from n[illegible]al burn patients' medical record formats (patient card) available in medical record unit of public hospital at North shewa zone. All charts of burn patients with the diagnosed of burn in between January 1st 2018 to December 30th, 2022 at selective public hospital in north shewa were reviewed from patient

registries. The checklists well prepared in English language, a language in which burn patients' medical record/patient card was written in. It contains information on socio-demographic conditions, clinical factors and health related factors. Data were extracted from patient medical record via record-review.

Data quality control and assurance management

Pre-test were done on 5% of the study samples on Hakim Gizaw hospital, is assumed to have similar characteristics to the study area, after the pre-test necessary correction was done on the checklist to amend language translation error and coherence of the checklist. Close supervision was conducte[illegible]y principal investigator and one MPH in Epidemiology expert who had exposures and experience in the study setting. Data collectors and supervisors were trained for three days [illegible]ethics, data collection tool and how to review the medical records and handle the patient card itself. Data collection well supervised on daily basis. Fille[illegible]ta were checked daily by supervisors and principal investigator to m[illegible]ize the errors that was created during review as early as possible. Data collectors were review [illegible]ecorded card in a separate place to keep privacy and confidentiality information.

Data Analysis procedures

The collected data were checked manually for any incompleteness and inconsistency. Data were entered using Epi-Data version 4.6 and analyzed using SPSS version 26. Descriptive statistics (frequency and percentage) for categorical predictors were done. Statistical association between each independent variable with dependent variable was assessed using bivariable logistics regression. In the bivariable analysis, a[illegible]edictor with P-value<0.25 were consid[illegible] for multivariable analysis. Model of fitness was assessed by Hosmer-Lemeshow test was not significant (0.212). The[illegible]linearitiy among independent variable were assessed by Variance inflation factor (1-2.5) those variables with a VIF of less than 10 were considered as no multi-collinearitiy. Multivariable logistic regression analysis was conducted to identify factors significantly associated with outcome of burn injury. Significance of association was declared at a p-value of ≤ 0.05 within 95% CI.

Result

Socio-demographic characteristics

A total of 408 participants participated with response rat[illegible]97%. Among 408 participants 44.9 % were male. From the total of study participant (n=252) 68.3% were single, and 30.1% were

married. Most of them were government [illegible]loyed and students. Most of the participant was at home when burn happened seen in table 1.

Table 1: Socio-demography characteristics of participants at public Hospitals in north shewa zone amhara region Ethiopia 2023 (n=408)

Variables	Category	Frequency	Percent
Sex	Male	183	44.9
	Female	225	55.1
Age of participant	≤15 year	208	51.0
	16-40 year	174	42.6
	≥41 year	26	6.4
Marital status(n=252)	Single	172	68.3
	Married	75	30.1
	Widowed	5	1.6
Occupation(n=254)	Gove[illegible]ent employed	54	21.5
	NGO	44	17.5
	Merchant	27	10.8
	Farmer	28	11.6
	Daily laborer	5	1.6
	House wife	41	16.3
	Student	52	20.7
Where a patient was found when burn occurred	Home	365	89.5
	Street	8	2
	School	28	6.9
	Work place	7	1.7

Health relate[illegible]tors

In this study the main circumstances of surrounding burn 98.8% participants were through accident and the remaining 1.8 % was assault. Most of the participant have bee[illegible]perficial thickness [illegible] (71.6%), 27.5% participants had partial thickness burn and only 1% participants have been full thickness burn injury. From the participant 5.1% were having been comorbidity with Epilepsy. Among the study participants 10.3[illegible] had pre-hospital intervention described as table 2.

Table 2: Health[illegible]ated factors of participants at public Hospitals in north shewa zone amhara region Ethiopia 2023(n=408)

**Variables**	**Category**	**Frequency**	**Percent**
The main circumstance of surrounding burn	Accident	403	98.8
	Assault	5	1.8
How was the depth of burn injury	Superficial	292	71.6
	Partial thickness	107	27.5
	Full thickness	9	1

How much body part affected via burn	≤20%<br>≥21%	319<br>89	79<br>21
Did your neck and head affected	Yes<br>No	168<br>240	57.6<br>42.4
Did your upper extremity affected	Yes<br>No	161<br>247	39.4<br>60.6
Did your lower extremity affected	Yes<br>No	64<br>344	15.7<br>84.3
Did your anterior trunk affected	Yes<br>No	9<br>399	2.2<br>97.8
Did you have comorbidity (Epilepsy)	Yes<br>No	21<br>387	5.1<br>94.9
Did you have pre- hospital intervention	Yes<br>No	42<br>362	11.3<br>88.7

Clinical characteristics of participants

From the participant admitted in hospital 75.2% were staying less than fifteen day. Participants during hospital stay 22.1% were took fluid and electrolyte, 22.3% were have been [illegible]nd management and most of them were [illegible]l antibiotic. From the participants their outcome of burn injury with complication 15.1% had disability, 20.5 death and 64.4 [illegible]re have been scaring as shown in table 3.

Table 3: clinical factors of participants at public Hospitals in north shewa zone amhara region Ethiopia 2023(n=408).

**Variables**	**Category**	**Frequency**	**Percent**
Did you used fluid and electrolyte	Yes<br>No	90<br>318	22.1<br>77.9
Did you used burn wound management	Yes<br>No	91<br>317	22.3<br>77.7
Did you used antibiotic	Yes<br>No	370<br>38	90.7<br>9.3
Did you used pain management	Yes<br>No	326<br>82	79.9<br>20.1
Length of stay in Hospital(n=303)	≤ 15day<br>16-25 day<br>≥26 day	228<br>25<br>50	75.2<br>8.3<br>16.5
If burn outcome with complication (n=146)	Death<br>Disability<br>Scaring	30<br>22<br>94	20.5<br>15.1<br>64.4

C[illegible] of burn

In this study showed that flame (35.5%) were highly cause of burn injury, scalds (27.8%) were the second cause of burn, steam (13.2%) were the third, chemical (10.7%) burn were the fourth and electrical (12.8%) were the last cause of burn as seen in Figure 3.

Outcome of burn injury

In the study, the proportion of the outcome of burn injury discharge[illegible]th complication was 40[illegible](95% CI: 36.5– 45.6) (fig 4). The main adverse complications burn injury were 12 (8%) died, 22(15.3%) disability and 112 (76.7) scar forming as described as Figure 4.

Multivariate logistic regression

Data were analyzed using binary logistic regression analysis. Statistical associations were checked by 95% CI and odds ratio. Those variables which had a p-value less than 0.25 in the binary logistical regression analysis were eligible for multivariabl[illegible]gistic regressions. Finally, the adjusted odds ratio was checked and the significant variables p value<0.05 were considered as associated factors for burn outcome of discharged with complication.

Patients who had pre-hospital intervention was [illegible]ly four (AOR= 3.8, 95% CI, 1.11-13.25) times more likely to develop[illegible]charged with complication compared to patients who have no[illegible] pre-hospital intervention. Similarly, this study revealed that the outcome of burn injury discharged with complication was (AOR= 4.3, 95% CI, 1.52-12.32) times more likely burn in scalds compared to patients with electrical, chemical and flame burn

Patients who had provided fluid and electrolyte were 76% less likely to the outcome of burn injury discharged with complication than patients who ha[illegible] use fluid and electrolyte (AOR= 0.24, 95%CI, 0.10-0.56). Similarly, the occurrence of TBS area less than twenty percent burned were 66[illegible]s likely to the outcome of burn injury discharged with complication compared to patients TBS area burned greater than twenty one percent (AOR= 0.34, 95%CI, 0.14-0.82) as shown in table 4.

Table 4[illegible]variable and multivariable logistic regression on poor outcome of burn injury among burn patients attending at public Hospitals in north shewa zone amhara region Ethiopia 2023(n=408).

Variables		**Outcome of burn injury**				
		Discharged with complication	***Discharged without complication***	COR (95% CI)	AOR(95% CI)	P-value
Age of participant	≤15 year	128	80	1.86(1.82-4.23)	2.57(2.52-12.59)	0.24
	16-40 year	101	73	1.6(1.70-3.69)	3.52(4.75-16.35)	0.10
	≥41 year	12	14	1		
Length of hospital stay	≤15 day	117	111	0.59(0.31-1.11)	0.72(0.35-1.49)	0.38
	16-25 day	20	5	2.25(1.72-7.01)	2.28(1.64-8.14)	0.20
	≥ 26 day	32	18	1		

What was the main cause of burn injury	Flame<br>Scald<br>Steam<br>Chemical<br>Electrical	92 51<br>73 38<br>25 29<br>21 22<br>26 26	1.80(1.94-3.42)<br>1.94(1.99-3.80)<br>0.82(0.38-1.78)<br>0.95(0.42-2.14)<br>1	1.13(1.55-3.46)<br>4.33(1.52-12.32)<br>1.80(0.50-6.49)<br>0.62(0.18-2.08)	0.49<br>**0.006***<br>0.36<br>0.44
TBS in %	≤20%<br>≥21	42 40<br>199 118	0.62(0.38-1.06)<br>1	0.34(0.14-0.82)	**0.017***
Sex of participants	Male<br>Female	114 69<br>127 98	1.2(1.85-1.89)<br>1	1.17(1.65-2.09)	0.58
Did you used antibiotic	Yes<br>No	214 156<br>27 11	0.55(0.26-1.16)<br>1	0.56(0.16-1.90)	0.35
Did you used fluid and electrolyte	Yes<br>No	37 53<br>204 114	0.39(0.24-0.62)<br>1	0.24(0.10-0.56)	**0.001***
Did you have pre-hospital intervention	Yes<br>No	19 22<br>218 145	0.57(0.30-1.09)<br>1	3.84(1.11-13.25)	**0.033***
Was your lower extremity affected	Yes<br>No	42 23<br>199 144	1.32(1.76-2.29)<br>1	3.38(1.96-11.88)	0.29
Was your upper extremity affected	Yes<br>No	128 107<br>113 60	0.66(0.44-0.99)<br>1	0.98(0.48-2.00)	0.96
Did you have co-morbidity(Epilepsy)	Yes<br>No	15 6<br>226 161	1.78(1.67-4.68)<br>1	0.71(1.17-2.98)	0.64

Discussion

In this study 40.9% (95% CI: 36.5– 45.6) was the outcome of burn injury discharged with complication. Having pre-hospital intervention, patients with scalds burn, early fluid and electrolyte provided and having total body surface area <20% were significant factors [illegible]ciated with discharged with complication. In this study, the proportion of the outcome of burn injury discharged with complication was 40.9% (95% CI: 36.5– 45.6). The result of this finding high compared to studies in Ayder referral hospital, Mekelle city, Tigria regional state and Addis Abeba Yekatit 12 Hospital, Ethiopia, 1[illegible]% and 17.3% were poor outcome of burn injury [32, 33]. The possible reason might be due to study area, dated of time and the sampling unit. Another similar study in Addis Ababa burn, emergency and trauma hospital, the proportion of outcome of burn injury only 14.2% were discharged with complication [34]. The difference of this might be, in the burn, emergency and trauma hospital had more sophisticated equipment at emergency intensive care unit, had enough emergency care health professionals and has well equipped infrastructure at health institution specifically for burn injury patients, it might be a reason for low proportion of b[illegible]d patients with the outcome of burn injury discharged with complication. But, this finding consistent with study done in Haromia University [35]. Another

stu[illegible] conducted in Tanzania and Nepal revealed different result [36] [37]. The reason might be duo to study population, area of study, period and time of data collection could be a reason for this difference. In this study the most frequent cause of injury is flame which accounts for 35.5% followed by scalds, steam, electrical and chemical burn. The result of this study congruent wi[illegible] studies done in Gonder, Ethiopia and Tanzania [36, 38]. But this study contradicts to studies in Addis Ababa burn, emergency and trauma hospital and Ayder referral hospital, Mekelle city [32, 34]. The possible justification might be due to population distribution at study area and cultural variation between populations to population. In this study flame burn is the number one cause for the study population followed by scalds, steams, electrical and chemical burns.

In this finding the affected body surface area (TBSA (%) due to burn injury is an important feature in the determination of the burn injury. From the study participants 79% patients had burn extent of less than 20% of TBSA. Finding of this study revealed that the occurrence of TBS area less than twenty percent burned were 66% less likely to discharge with compilation as compared to patients TBS area burned greater than twenty-one percent. Which is in line with the study showed in Bahir Dar [33]. This marks in resulting to decline intravascular hypervolemia which can produce complication like ischemia to body organs. Similarly, patients who had provided fluid and electrolyte were 76% less likely to the outcome of burn injury discharged with compli[illegible]n than patients who had not use fluid and electrolyte during hospital stay. The finding also congruent with study done in Ayder referral hospital, Mekelle city [32]. This could implicate that increasing public awareness regarding health seeking behavi[illegible]nd early admission for intera-veins infusion can diminish complication. This confirmed that patients established timely supportive interventions, related to fluid and electrolyte resulting in fewer burn complications. Patients who had pre-hospital intervention were three point eight times more likely the outcome of burn injury discharged with complication then patients who have not pre-hospital intervention. This finding was not equivalent [illegible]h studies conducted in Haromia and Tanzania [35] ,[36]. The reason might be due to the difference of preexisting medical condition before arriving at health institution, extent and depth of burn injury, cultural tradition among burn management and quality of care in the community. Patients who sustained scald burn were four point three times more likely the outcome of burn injury discharged [illegible] complication as compared to patients with electrical, chemical and flame burn. This finding supported with study conducted in South Gondar zone government hospitals, Ethiopia [38].But this result different from study done in Buganda Medical Centre in Northwestern Tanzania, that showed flame, electrical and

chemical burn were more likely complication as compared to scald burn [36]. The difference might be duo to sample size, study population, life style and socio-cultural because of those cause which are prone to different burn injury.

Conclusion

This study demonstrates a significantly higher level of the outcome of burn injury discharged with complication, leading to increased death and other worst outcomes. Having p[illegible]ospital intervention and the cause of burn with scalds were significant factors associated with discharged with complication. Early flui[illegible]d electrolyte provided and having TBS area less than twenty percent as a protective effect for patients with poor outcome of burn injury.

Recommendation

To health professionals

- -Encourage health education on pre-hospital intervention to prevent and perform initial intervention for patients with burn injury. Increase the awareness and impact of scald burn for clients as well as the community.
- -Focus to providing fluid and electrolyte for patients need to hospital admission and their total body surface area affected more than twenty percent.

To the regional health office

- - There should be well equipped burn units to reduce poor outcome of burn injury.

To researchers

- - Recommend for further case control studies that determine the risk factor, for poor outcome of burn injuries.

Limitation of the study

This study has a number of restrictions. The first drawback is that a few variables are absent from the typical burn registration books. Unable to determine the precise number of burn patients who were hospitalized or discharged because the data is retrospective.

Acronyms and abbreviations

AIDS	Acquired Immune Deficiency Syndrome
AOR	Adjusted Odd Ratio
BCC	Burn Care Center
BSA	Body Surface Area
DALY	Disability-Adjusted Life-Years
IVF	Intravenous Fluid

MRU	Medical Record Unit
PI	Principal Investigator
RTA	Road Traffic Accident
SPSS	Statistical Package for Social Science
TBSA	Total Body Surface Area
WHO	World Health Organization

Ethical Approval

Ethical approval and clearance were obtained from the institutional review board (IRB) of Asrat Woldeyes Health Science Campus Permission was obtained from Zonal health office, and selected hospital chief-executive officer. All aspects of basic ethical research principles were addressed, so the study participants were selected based on the research requirements. The study participant name was not be written in the questionaries' form and never be used in connection with any information you tell us. All information given by the study participant was kept strictly confidential. Codes and aggregate reporting were used to eliminate names and other personal identifiers of respondents throughout the study process to ensure anonymity.

Funding: The authors have not received specific funding for this work.

Competing interests: The authors declare that they have no competing interests exist.

Author's contributions

Conceptualization: Ejigu Mulugeta.

Data curation: Ejigu Mulugeta.

Formal analysis: Ejigu Mulugeta.

Investigation: Ejigu Mulugeta, Akine Eshete, Mekasha Getnet, Dr. Abebe Minda, Abebe Nigussie

Methodology: Ejigu Mulugeta, Akine Eshete, Mekasha Getnet, Dr. Abebe Minda, Abebe Nigussie

Project administration: Ejigu Mulugeta, Akine Eshete, Mekasha Getnet, Dr. Abebe Minda, Abebe Nigussie

Resources: Ejigu Mulugeta, Akine Eshete, Mekasha Getnet, Dr. Abebe Minda, Abebe Nigussie

Software: Ejigu Mulugeta, Akine Eshete, Mekasha Getnet, Dr. Abebe Minda, Abebe Nigussie

Supervision: Ejigu Mulugeta, Akine Eshete, Mekasha Getnet, Dr. Abebe Minda, Abebe Nigussie

Validation: Ejigu Mulugeta, Akine Eshete, Mekasha Getnet, Dr. Abebe Minda, Abebe Nigussie

Visualization: Ejigu Mulugeta, Akine Eshete, Mekasha Getnet, Dr. Abebe Minda, Abebe Nigussie.

Writing – Ejigu Mulugeta, Akine Eshete, Mekasha Getnet, Dr. Abebe Minda, Abebe Nigussie

Writing – review & editing: Ejigu Mulugeta, Akine Eshete, Mekasha Getnet, Dr. Abebe Minda ,Abebe Nigussie

Acknowledgments

We would like to thank the study participants, data collectors, and supervisors who were involved in this study and spent their valuable time responding to my study.

Reference

1. de Figueiredo, T.B., et al., *Mobilization practices for patients with burn injury in critical care.* Burns, 2020. **46**(2): p. 314-321.
2. Vermaak, P.V., et al., *Thromboprophylaxis in adult and paediatric burn patients: a survey of practice in the United Kingdom.* Burns, 2019. **45**(6): p. 1379-1385.
3. Biru, W.J. and F.T. Mekonnen, *Epidemiology and outcome of childhood burn injury in Hawassa University Comprehensive Specialized Hospital, Hawassa, Southern Ethiopia.* Indian Journal of Burns, 2020. **28**(1): p. 51.
4. Heydarikhayat, N., et al., *Effect of post-hospital discharge follow-up on health status in patients with burn injuries: a randomized clinical trial.* International journal of community based nursing and midwifery, 2018. **6**(4): p. 293.
5. Karlie, J. and A. Wardhana, *External Validation of Belgian Outcome of Burn Injury Score on Burned Patient In Burn Unit dr. Cipto Mangunkusumo General Hospital.* The New Ropanasuri Journal of Surgery, 2017. **2**(1): p. 3.
6. Theodorakopoulou, E., et al., *London calling Gaza: The role of international collaborations in the globalisation of postgraduate burn care education.* Scars, Burns & Healing, 2019. **5**: p. 2059513119830519.
7. Tupper, C. and A. Doyal, *OSHA Fire Safety*, in *StatPearls [Internet]*. 2022, StatPearls Publishing.
8. Gonçalves, N., et al., *A randomized controlled trial of an educational programme with telephone reinforcement to improve perceived health status of Brazilian burn victims at 6-month post discharge.* Journal of Advanced Nursing, 2016. **72**(10): p. 2508-2523.

9. James, S.L., et al., *Epidemiology of injuries from fire, heat and hot substances: global, regional and national morbidity and mortality estimates from the Global Burden of Disease 2017 study.* Injury prevention, 2020. **26**(Suppl 2): p. i36-i45.
10. Sadeghian, F., et al., *Mortality and years of life lost due to burn injury among older Iranian people; a cross-sectional study.* Archives of academic emergency medicine, 2022. **10**(1).
11. Botman, M., et al., *Surgical burn care in sub-Saharan Africa: a systematic review.* Burns Open, 2019. **3**(4): p. 129-134.
12. Mulatu, D., et al., *Outcome of burn injury and associated factor among patient visited at Addis Ababa burn, emergency and trauma hospital: a two years hospital-based cross-sectional study.* BMC emergency medicine, 2022. **22**(1): p. 1-14.
13. Gessesse, F.G., *Retrospective Study on Outcome of Burn Injury and Associated Factors among Children at Felegehiwot Referral Hospital, Bahirdar Town, Amhara Regional State, Ethiopia, 2017*. 2017, Addis Ababa University.
14. Gendy, J.F., *Prediction of Risk Factors for Infection Occurrence in Patients with Burn Injury.* Journal of Biology, Agriculture and Healthcare, 2017. **7**(12): p. 22-30.
15. Biswas, R., et al., *A study on treatment profile and covariates of outcome of burn injury in a Tertiary Medical hospital in West Bengal.*
16. Mehanna, A.F., *Effect of Distraction Techniques on Minimizing Pain Associated With Burn Dressing Changes among Preschool Children.*
17. Chalie, M., *Magnitude of Mortality and its Associated factors Among Burn Victim Pediatrics Population Admitted to South Gondar Zone Public Hospitals, Amhara Regional State, Ethiopia, 2020*. 2020.
18. Fadeyibi, I.O., et al., *Practice of first aid in burn related injuries in a developing country.* Burns, 2015. **41**(6): p. 1322-1332.
19. Elsherbiny, O.E., et al., *Effect of burn rehabilitation program on improving quality of life (QoL) for hand burns patients: a randomized controlled study.* European Journal of Plastic Surgery, 2018. **41**: p. 451-458.
20. Keshri, V.R. and J. Jagnoor, *Burns in India: a call for health policy action.* The Lancet Public Health, 2022. **7**(1): p. e8-e9.
21. Edwards, J. and P. Buckley, *Who's the Most at Risk of Work-Related Violence?* Occupational Medicine, 2022. **72**(1): p. 25-27.

22. Koyro, K.I., et al., *Burn Guidelines—An International Comparison.* European Burn Journal, 2021. **2**(3): p. 125-139.
23. Tian, H., et al., *Epidemiology and outcome analysis of facial burns: A retrospective multicentre study 2011–2015.* Burns, 2020. **46**(3): p. 718-726.
24. Evans, C.S., et al., *Burn related injuries: a nationwide analysis of adult inter-facility transfers over a six-year period in the United States.* BMC emergency medicine, 2022. **22**(1): p. 1-9.
25. Cleary, M., et al., *The importance of mental health considerations for critical care burns patients.* Journal of Advanced Nursing, 2018. **74**(6): p. 1233-1235.
26. Ferraris, D.M., *Speech and language therapy for burn injuries in South Africa: knowledge and experience, evidence-based involvement, access, referral pathways, and training and education needs*. 2021, Stellenbosch: Stellenbosch University.
27. Barnett, B.S., et al., *Qualitative analysis of a psychological supportive counseling group for burn survivors and families in Malawi.* Burns, 2017. **43**(3): p. 602-607.
28. Amakobe, N.C., *Factors associated with increasing incidence of burns among adult patients admitted in the Kenyatta National Hospital Burns Unit.* Journal of Health, Medicine and Nursing, 2017. **1**(2): p. 60-84.
29. Stokes, M. and W. Johnson, *Burns in the Third World: an unmet need.* Annals of burns and fire disasters, 2017. **30**(4): p. 243.
30. Chung, K.K., et al., *Renal replacement therapy in severe burns: a multicenter observational study.* Journal of Burn Care & Research, 2018. **39**(6): p. 1017-1021.
31. Alemayehu, S., et al., *Management outcome of burn injury and associated factors among hospitalized children at Ayder Referral Hospital, Tigray, Ethiopia.* International journal of pediatrics, 2020. **2020**.
32. Alemayehu, S., et al., *Management Outcome of Burn Injury and Associated Factors among Hospitalized Children at Ayder Referral Hospital, Tigray, Ethiopia.* Int J Pediatr, 2020. **2020**: p. 9136256.
33. *Retrospective study on outcome of burn injury and associated factors among children at felegehiwot referral hospital, bahirdar town, amhara regional state, ethiopia.*
34. Mulatu, D., et al., *Outcome of burn injury and associated factor among patient visited at Addis Ababa burn, emergency and trauma hospital: a two years hospital-based cross-sectional study.* BMC Emerg Med, 2022. **22**(1): p. 199.

35. *Outcome of burn injury and associated factors among burn patients attending hiwot fana speciallized university hospital, eastern ethiopia.*
36. *Pattern of childhood burn injuries and theirmanagement outcome at Bugando Medical Centre in Northwestern Tanzania.*
37. Khatiwada, S., S. Adhikari, and B.N.A. Sudhin, *Epidemiology and outcome of acute burn patients at a new dedicated burn centre in Nepal.* Journal of Society of Surgeons of Nepal, 2020. **23**(1): p. 19-29.
38. Tiruneh, C.M., et al., *Magnitude of mortality and its associated factors among Burn victim children admitted to South Gondar zone government hospitals, Ethiopia, from 2015 to 2019.* Ital J Pediatr, 2022. **48**(1): p. 12.

Figure Click here to access/download;Figure;figure.docx

Conceptual frame work

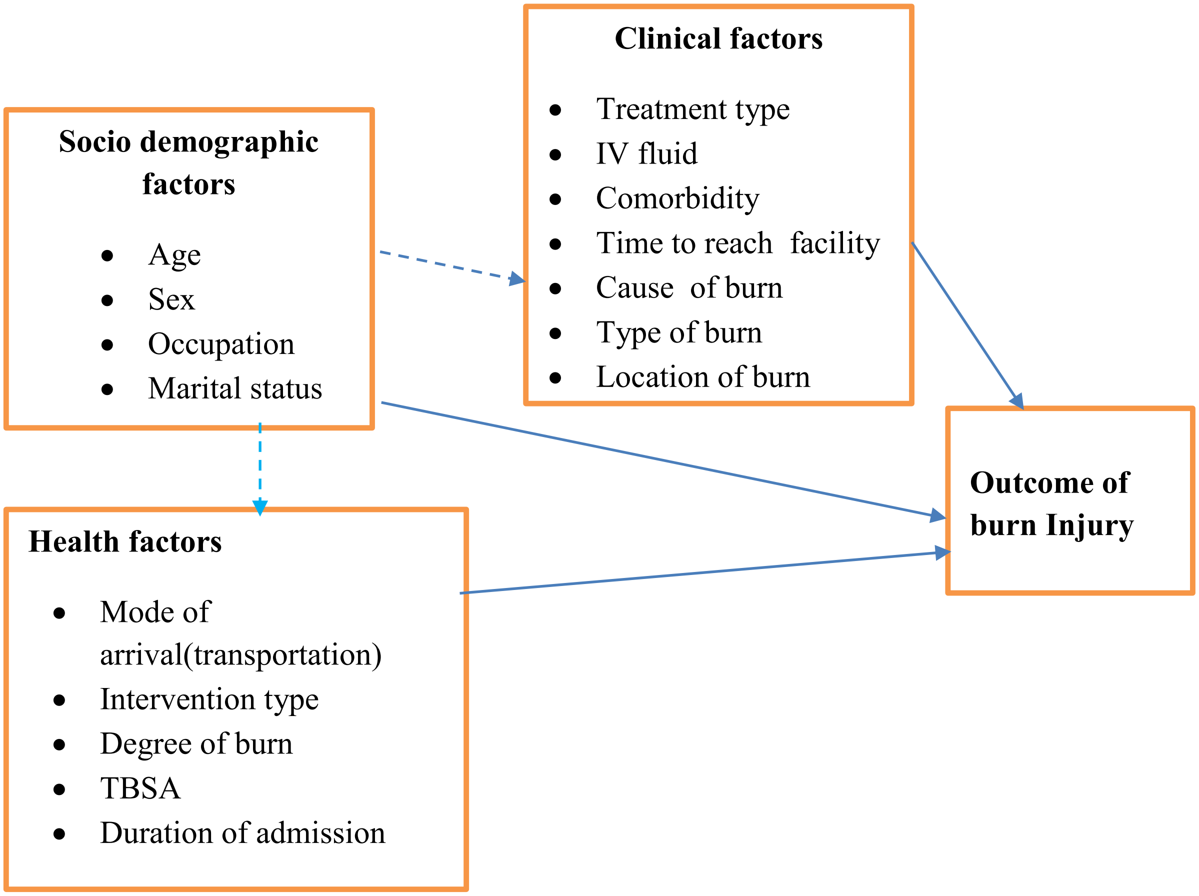

Figure 1:- Conceptual frame work for a study on the outcome of burn injury and its associated factors among burn patient at public hospital in North shewa zone, 2023 [1-4].

Sampling techniques

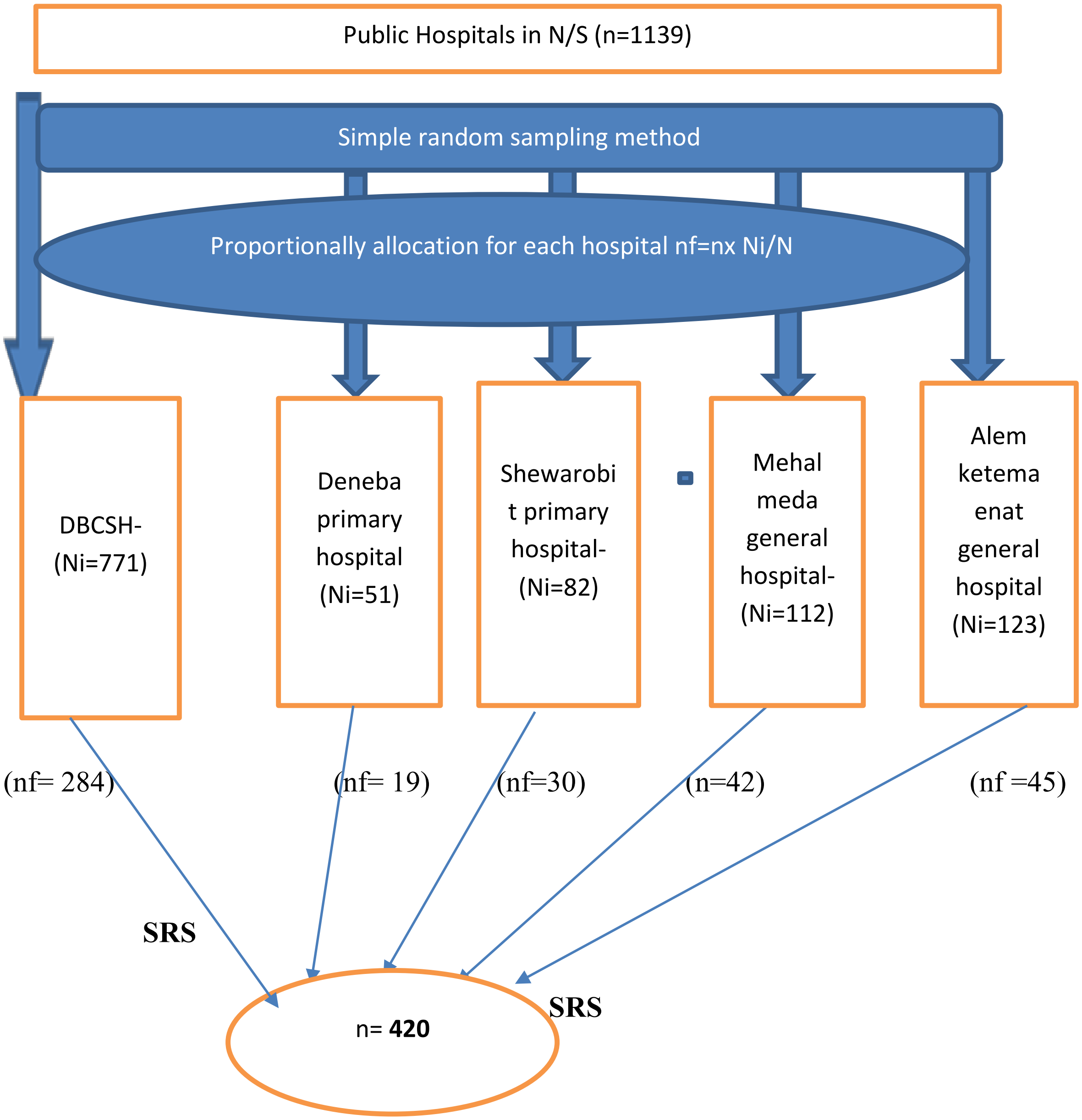

Figure 2:- Schematic presentation of sampling procedure for a study on the outcome of burn injury and its associated factors among burn patient at public hospital in North shewa zone, 2023

Cause of burn injury

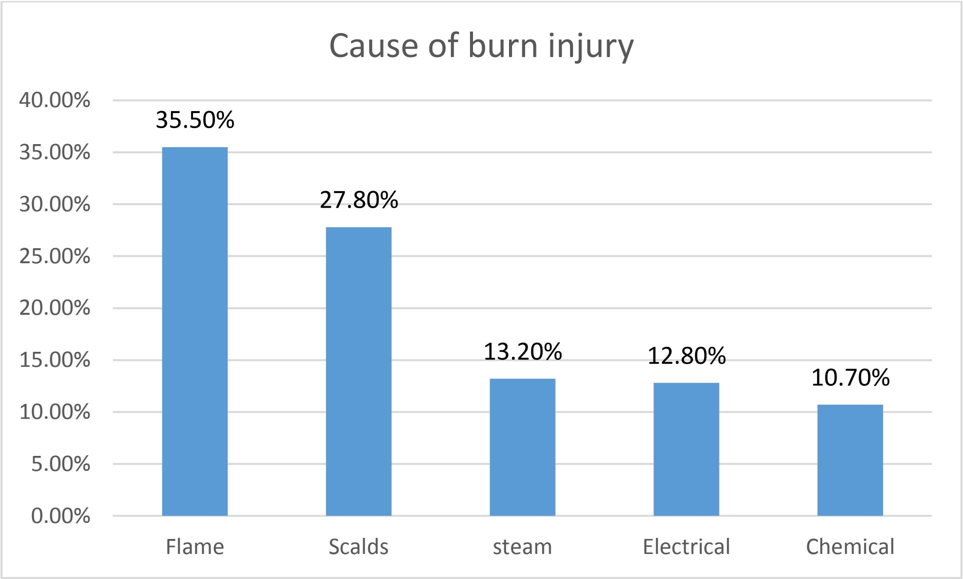

Figure 3 Schematic presentation of cause of burn among burn patients attending at public Hospitals in north shewa zone Amhara region Ethiopia 2023(n=408).

Outcome of burn injury

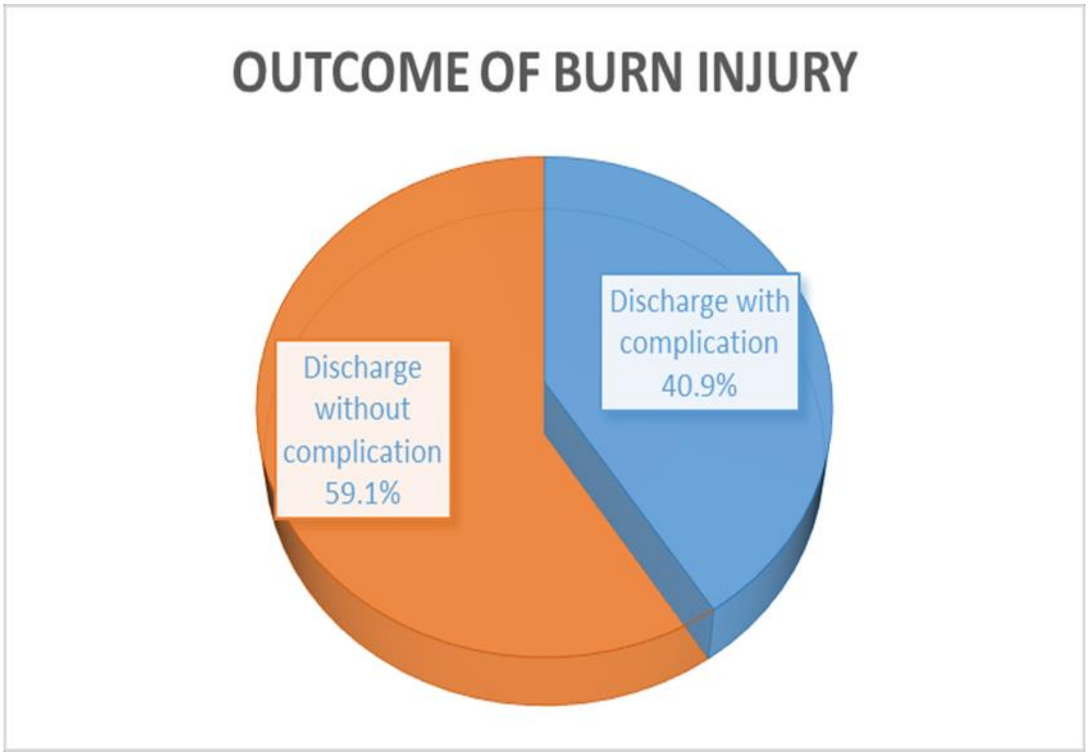

Figure 4 Schematic presentation of the proportion on poor outcome among burn patients attending at public Hospitals in north shewa zone amhara region Ethiopia 2023(n=408).

Click here to access/download
Supporting Information
supporting file.docx

Click here to access/download
Supporting Information
AnyScanner_08_02_2023.pdf

Letter of response

To all honorable reviewers and editors

Dear Reviewers and Editors, We are very appreciative for your relevant comments, questions and recommendations. Following this, we have provided our responses point by point as presented below.

Authors Responses

1. Where possible, we encourage authors to provide relevant data underlying their research with their submission in order to facilitate editorial and peer review, even if the data may be restricted from publication or made private by a data repository prior to article acceptance. Many repositories permit private access for review purposes, and have policies for public release at publication. For data concerning human research subjects, please anonymize the data as appropriate and follow pertinent legal and ethical protocols prior to sharing with editors and reviewers. If you are unable to share any pertinent data at the time of editorial and peer review, please kindly let the journal office know.

Answer

We accept and amended it accordingly

2. Your Cover Letter indicates this submission is for the [Outcome of burn injury] Collection, but this is not a current Collection at PLOS. We will proceed with editor assignment, but please let us know if you meant to select a different Collection.

   Answer

   We have study focus on burn outcome injury (Discharged with complication or without complication) but not collection

3. Thank you for providing a cover letter. Please ensure that your cover letter is addressed specifically to PLOS Global Public Health

   Answer

   We accept and amended it accordingly

4. Please complete all items on the Human Participants Research Checklist that are relevant for yoursubmission,byfollowingthislink:http://journals.plos.org/plosone/s/file?id=dc11/PLOS%20Human%20Participants%20Research%20Checklist.docx. There may be overlap between the checklist items and other queries listed here; please address any duplicated queries both in your response email and on the checklist itself. Upload the completed Human Participants Research checklist as file type “Other” when you re-submit your manuscript. This document

is for internal journal use only and will not be published if your article is accepted. The requested information will help us to assess whether your submission complies with PLOS's policies and adheres to applicable reporting standards. Note that your manuscript may be rejected if you provide incomplete or inadequate responses to the checklist questions and that changing the 'Section/Category' of your article does not affect this requirement.

Answer

We accept and amended it accordingly

5. Your study includes child participants; please update your Ethics Statement with confirmation that written informed consent was obtained from the parent/guardian of each participant under 18 years of age.

   Answer

We are appreciating your helpful advice. We did not use the questions asked during the interview; instead, we used secondary data from the documentation (review chart). Consequently, we did not get children's informed consent.
