# Supplementary material for: Outcome of burn injury and its associated factors among burn patients attending public hospitals in North Showa Zone, Ethiopia: A cross-sectional study
Source: PLOS Glob Public Health. 2024 Sep 16;4(9):e0003682. doi: 10.1371/journal.pgph.0003682 (PMC11404801; doi:10.1371/journal.pgph.0003682)
Supplement: Attachment — Submitted filename: PGPH-D-23-01362_R1_reviewer.pdf [file pgph.0003682.s006.pdf]

Manuscript Click here to access/download;Manuscript;Manscript.docx

Outcome of Burn Injury and its Associated Factors among Burn Patients Attending Public Hospitals in North Showa Zone, Ethiopia: A Cross-Sectional Study

Ejigu Mulugeta Shewaye[1], Akine Eshete Abosetugn[2], Mekasha Getnet[3], Dr. Abebe Minda [4], Abebe Nigussie Ayele [5],Mitiku tefera[6]

Address

Email; ejigu.mulugeta1234@gmail.com

[1]Departments of Nursing, Debre Berhan University, Ethiopia,

Email; _akine.eshete@yahoo.com

[2] Department of Nursing, Debre Berhan University, Asrate Woldeyes Health Science Campus, Ethiopia

Email; mekashagetnet@gmail.com

[3]Department of Nursing, Debre Berhan University, Asrate Woldeyes Health Science Campus, Ethiopia

Email: abebe2014nigussie@gmail.com

[4]Department of Nursing, Debre Berhan University, Asrate Woldeyes Health Science Campus, Ethiopia

Email: abe.minda@yahoo.com

[5]Department of Nursing, Debre Berhan University, Asrate Woldeyes Health Science Campus, Ethiopia

mitikutefera2632@gmail.com

[6] Department of Midwifery Debre berhan health science College, Amhara Ethiopia.

Abstract

Background: Burn injury is a major contributor to morbidity and mortality in Sub-Saharan Africa, including Ethiopia, and requires regular interventions. There is limited evidence on the outcome of burn injury status in Ethiopia, including the study area.

Objective: To assess the outcome of burn injury and associated factors among burn patients attending public Hospital in North Showa zone, Ethiopia.2023

Method: an institution-based cross-sectional study was conducted among 420 burn patients in a public hospital in the North Showa zone from April to May 2023. Systematic random sampling was used to select patients' cards. Structured checklists were used to extract data from burn patients' medical records. Data were entered using Epi-data version 4.6 and analyzed using SPSS version 25. Variables with a p-value of ≤ 0.25 in the bivariable logistic regression were included in the multivariable logistic regression.

Result: In this study, 40.9% (95% CI: 36.5–45.6) were discharged with complications. The odds of developing complication among patients having pre-hospital intervention were n[illegible] four times the odds of not having the intervention (AOR = 3.8, 95% CI, 1.11–13.25), and The odds of developing complication among patients having scald were four times the odds of not having scalds (AOR = 4.3, 95% CI, 1.52-12.32). A patient who is received fluid and electrolytes were 76% less likely to develop the outcome of burn injury discharged with complication, and patients who received total body surface area burn 20% less likely to develop discharge with complication.

Conclusion: This study demonstrates a significantly higher level of outcome for patients with burn injuries who were disc[illegible]ed with complication, leading to death and other bad outcomes. Therefore, stake holders are more emphasis in health education on pre-hospital intervention, treatment of the cause of burns, and providing fluid and electrolytes for patients with burn injuries.

Keywords: Burn injury, burn patients, outcome, Ethiopia.

Introduction

Burn is a term used to describe skin and tissue damage brought on by a fire, scald, electrical shock, chemical burn, or radioactive radiation. Due to the prolonged hospitalization for rehabilitation and the treatment of wounds and scars, burns are the most expensive traumatic injuries[1]. Large burn victims have many of the same physiological reactions as victims of major trauma, particularly venous thromboembolism [2]. It is one of the frequent and unpleasant forms of trauma in addition to being a substantial worldwide health problem that increases morbidity and death and ca[illegible] significant material, psychological, and financial damage [3]. Burns are damaging injuries that are linked to morbidity, a reduction in quality of life, and emotional well-being [4]. Mortality and morbidity from burns remain high, particularly in developing countries [5]. These areas are also areas where the lack of socio-economic growth , and geopolitical instability create additional barriers not only to the delivery of health care, but also to the acquisition of continuous professional development [6]. Burns are estimated to be the root cause of 180,000 deaths worldwide, most of which currently occurs in low- and middle-income countries [7]. Burns are the fourth leading cause of trauma in the world and are common in developing countries [8]. Burn injury incidence and mortality affect all regions of the world, but are most concentrated in LMICs [9]. Burns are a major public health problem worldwide [10]. The fatality rate for child burn victims in Africans were 27.8 % [11].

A study shows that the magnitude of burns with complications in Ethiopia was 14.9% , the highest risk groups were children age groups compare to the elderly [12]. In almost all age groups, the prevalence of burns is higher in women [13]. The increase in burn risk in 3rd degree burns was more likely in case of wound infection and the risk of such infection was also considered in burn surgery [14]. Wound infection and septicemia were the two most common complications in burn patients [15]. A burn injury contains both nociceptive and neuropathic pain components, so managing a person who suffer from burn injury is one of the most difficult challenges for the first responders [16]. Treating a burn requires many hours of wound care by nursing staff, possibly multiple surgical procedures, and expensive hospitalization [17]. Burns are the most painful and require special attention and are considered a major public health problem [18]. Burns are the fourth most common type of trauma causing death and disability after traffic accidents, falls and interpersonal violence [19]. Globally, burns are one of the leading causes of disability, accounting for more than 8 million disability-adjusted life years (DALYs) [20]. These costs of burn injury are from high income settings. The authors may consider replacing them with similar evidence from low-resource settings which may be more relevant to the setting of this study. In South Africa, an estimate of US$26 million is spent annually on burn injury, but indirect costs such as lost wages, long-term treatment for disfigurement and emotional trauma, and the contribution of family resources also contribute to socioeconomic impacts indirectly [21]. Currently, most burn centers around the world care for patients based on a protocol of burn management like emergency phase management, acute wound care and rehabilitation modalities [22]. In the 20th century, advances in burn care lead to an increased focus on reducing burn morbidity. [23]. Burns often result in significant psychological, educational and social stigma, and the resulting changes are exacerbated by many factors, including the circumstances of the burn, severity and location of the injury, personality characteristics of the injured person and access to supportive interpersonal communication, and social relations [24]. Both the patients and their family may experience physical and emotional suffering as a result of the burns and treatment [25]. Burns are the second most common injury in rural Nepal, accounting for 5 % admission in hospitals [7]. It is estimated that over a million people burn in Africa each year and 18% of hospitalizations are due to burns [26]. Burns are a common source of harm in developing countries including Ethiopia [27]. The incidence of burns varies by country and population group, and the severity of the burn depends on the degree of heat, duration of exposure, and thickness of the skin [28]. Burns occur mainly at home and in the

workplace, as in Bangladesh and Ethiopia, show that 80-90% of burns occur at home [29].In Ethiopia, the outcome of burn injury and associated factors among burn patients were not described. Hence, it needs more study to assess the possible outcome of burn and support uniform treatment protocol in health facility. Therefore, this study was assesses the outcome of burn injury and its associated factors a public hospital of North Shewa Zone, Amhara regional state, Ethiopia.

Methods

Study design, setting and period

An institution-based cross-sectional study was conducted among 420 burn patients in public Hospital in North Showa zone, Northeast, Ethiopia, 2023. Debre Berhan City is located 130-kilo meters from Addis Ababa, the capital city of Ethiopia and 696 km away from the Bahirdar city the Regional state of Amhara. The total population of North Showa Zone was 2,322,148 of whom those 1,171,510 are men and 1,150,638 women in 2019.According to Zonal Health Office Report, North Shewa has 164 private clinics, 97 governmental health centers, 391 health posts, 8 primary Hospitals of which two are private primary hos[illegible]s, 2 general hospitals, and one comprehensive specialized hospital from April to May, 2023. North Shewa Zone has a total of 10 public and 2 private hospitals.

Populations

Source Population: All patients who visited public hospital in North shoa zone, Ethiopia.

Study population: All burn patients who attended burn clinic have medical record folder in selected public hospital from the 1st of January 2018 to December 30th 2022 in North Showa zone, Amhara, Ethiopia.

Inclusion and Exclusion criteria

Inclusion criteria

All burn patients who attended North shewa zone public hospital, registered on patient card/medical record folders in hospital and his/her card/record was available in medical record unit/card room were included.

Exclusion criteria

Burn patients with incomplete records/information on either the burn or miss diagnosis upon review were excluded from the study.

Sample Size Determination and Sampling Techniques

Sample size for this study was determined by single population formulas. Sample size was calculated using the prevalence of burn injury , the prevalence in Addis Ababa (p=54.7%) [12] ,with 5% margin of error, 95% CI and 10% non-response rate were considered. By adding a 10% non-response rate, the total sample size becomes **420**.

There are 10 public hospitals in the North Showa Zone; from these, five were selected using a simple random sampling method. The medical record numbers (MRN) of the burn patient were used as a sampling frame. The sample was allocated proportionally for each hospital. Study participants were selected by using a systematic random sampling technique. First, determined the sampling interval (K) value by dividing the total admitted burn patient in the study period by the total sample size, which gives 2.17 ≈ 2 as shown in Figure 1.

Variables (seen in Figure 2)

Dependent variable

Outcome of Burn Injury discharged with complication or without complication

Independent variable

Socio demographic factors

Health factors

Clinical factors

Operational definitions

Discharge with complication: In this study the patients have been discharged with one of the following; like contracture, disfigurement, amputated, scar of skin graft and death as reported on the chart considered as poor outcome of burn injury.

Burn patients: A patient who sustained burn injury.

Burn injury: an injury of skin or other tissue caused by thermal, radiation, chemical or electrical contact [30].

Type of burn injury: classified by depth of skin layers as superficial, deep partial thickness, and full thickness and TBSA are involved.

Outcome of burn injury: when the patients admitted in hospital who diagnosed as discharged with complication or without complication as reported on the chart [31].

Data collection procedures

Data were collected using structured checklist adopted from national burn patients' medical record formats (patient card) available in medical record unit of the public hospital at North shewa zone. All charts of burn patients with the diagnosed of burn injury between January 1st 2018 to December 30th, 2022 at selective public hospital in north shewa zone were reviewed from patient registries. The checklists were well prepared in English language, a language in which burn patients' medical record/patient card was written in. It contains information on socio-demographic conditions, clinical factors and health related factors. Data were extracted from patient medical record via record-review.

Data quality control and assurance management

Pre-test were done on 5% of the study samples on Hakim Gizaw hospital, is assumed to have similar characteristics to the study area, after the pre-test necessary correction was done on the checklist to amend language translation error and coherence of the checklist. Close supervision was conducted by principal investigator and one MPH in Epidemiology expert who had experience in the study setting. Data collectors and supervisors were trained on ethics, data collection tool and how to review the medical records and handle the patient card itself. Data collection well supervised on daily basis. Filled data were checked daily by supervisors and principal investigator to minimize the errors that were created during review as early as possible. Data collectors were reviewed recorded card in a separate place to keep privacy and confidentiality information.

Data Analysis procedures

The collected data were checked manually for incompleteness and inconsistency. Data were entered using Epi-Data version 4.6 and analyzed using SPSS version 26. Descriptive statistics frequency and percentage for categorical predictors were done. Statistical association between each independent variable with dependent variable was assessed using bivariable logistics regression. In the bivariable analysis, a predictor with P-value<0.25 were considered for multivariable analysis. Goodness of fitness was assessed by Hosmer-Lemeshow test that was not significant (0.212). The collinearitiy among independent variable were assessed by Variance inflation factor (1-2.5), those variables with a VIF of less than 10 were considered as no multi-collinearitiy. Multivariable logistic regression analysis was conducted to identify factors significantly associated with outcome of burn injury. Significance of association was declared at a p-value of ≤ 0.05 within 95% CI.

Result
