# Supplementary material for: Outcome of burn injury and its associated factors among burn patients attending public hospitals in North Showa Zone, Ethiopia: A cross-sectional study
Source: PLOS Glob Public Health. 2024 Sep 16;4(9):e0003682. doi: 10.1371/journal.pgph.0003682 (PMC11404801; doi:10.1371/journal.pgph.0003682)
Supplement: Attachment — Submitted filename: Response to reviewers.docx [file pgph.0003682.s003.docx]

**Letter of response**

**To all honorable reviewers and editors**

Dear Reviewers and Editors, We are very appreciative for your relevant comments, questions and recommendations. Following this, we have provided our responses point by point as presented below.

**Authors Responses**

1. Where possible, we encourage authors to provide relevant data underlying their research with their submission in order to facilitate editorial and peer review, even if the data may be restricted from publication or made private by a data repository prior to article acceptance. Many repositories permit private access for review purposes, and have policies for public release at publication. For data concerning human research subjects, please anonymize the data as appropriate and follow pertinent legal and ethical protocols prior to sharing with editors and reviewers. If you are unable to share any pertinent data at the time of editorial and peer review, please kindly let the journal office know.

**Answer**

We accept and amended it accordingly

2. Your Cover Letter indicates this submission is for the [Outcome of burn injury] Collection, but this is not a current Collection at PLOS. We will proceed with editor assignment, but please let us know if you meant to select a different Collection.

**Answer**

**We have study focus on burn outcome injury (Discharged with complication or without complication) but not collection**

3. Thank you for providing a cover letter. Please ensure that your cover letter is addressed specifically to PLOS Global Public Health

**Answer**

**We accept and amended it accordingly**

4. Please complete all items on the Human Participants Research Checklist that are relevant for yoursubmission,byfollowingthislink:http://journals.plos.org/plosone/s/file?id=dc11/PLOS%20Human%20Participants%20Research%20Checklist.docx. There may be overlap between the checklist items and other queries listed here; please address any duplicated queries both in your response email and on the checklist itself. Upload the completed Human Participants Research checklist as file type “Other” when you re-submit your manuscript. This document is for internal journal use only and will not be published if your article is accepted. The requested information will help us to assess whether your submission complies with PLOS’s policies and adheres to applicable reporting standards. Note that your manuscript may be rejected if you provide incomplete or inadequate responses to the checklist questions and that changing the ‘Section/Category’ of your article does not affect this requirement.

**Answer**

**We accept and amended it accordingly**

5. Your study includes child participants; please update your Ethics Statement with confirmation that written informed consent was obtained from the parent/guardian of each participant under 18 years of age.

**Answer**

**We are appreciating your helpful advice. We did not use the questions asked during the interview; instead, we used secondary data from the documentation (review chart). Consequently, we did not get children's informed consent.**
