# Supplementary material for: Outcome of burn injury and its associated factors among burn patients attending public hospitals in North Showa Zone, Ethiopia: A cross-sectional study
Source: PLOS Glob Public Health. 2024 Sep 16;4(9):e0003682. doi: 10.1371/journal.pgph.0003682 (PMC11404801; doi:10.1371/journal.pgph.0003682)
Supplement: Attachment — Submitted filename: Response to reviewers.docx [file pgph.0003682.s005.docx]

**Letter of response**

**To all honorable reviewers and editors**

Dear Reviewers and Editors, We are very appreciative for your relevant comments, questions and recommendations. Following this, we have provided our responses point by point as presented below.

**Authors Responses**

1. Does this manuscript meet PLOS Global Public Health’s [publication criteria](https://journals.plos.org/globalpublichealth/s/criteria-for-publication)? Is the manuscript technically sound, and do the data support the conclusions? The manuscript must describe methodologically and ethically rigorous research with conclusions that are appropriately drawn based on the data presented.

Reviewer #1: Partly

Reviewer #2: Partly

**Answer**

We accept and amended it accordingly.

2. Has the statistical analysis been performed appropriately and rigorously?

Reviewer #1: Yes

Reviewer #2: I don't know

**Answer for Reviewer 2**

**We are appreciating for your view and thought fully review of our manuscript. But our outcome variable is categorical and binary; we use binary logistic regression analysis methode. And also before analysis, we checked Goodness of fitness was assessed by Hosmer-Lemeshow test & interpret in rigorously.**

3. Have the authors made all data underlying the findings in their manuscript fully available (please refer to the Data Availability Statement at the start of the manuscript PDF file)?

The [PLOS Data policy](https://journals.plos.org/globalpublichealth/s/data-availability) requires authors to make all data underlying the findings described in their manuscript fully available without restriction, with rare exception. The data should be provided as part of the manuscript or its supporting information, or deposited to a public repository. For example, in addition to summary statistics, the data points behind means, medians and variance measures should be available. If there are restrictions on publicly sharing data—e.g. participant privacy or use of data from a third party—those must be specified.

Reviewer #1: Yes

Reviewer #2: Yes

**Answer: N/A**

4. Is the manuscript presented in an intelligible fashion and written in Standard English?

PLOS Global Public Health does not copyedit accepted manuscripts, so the language in submitted articles must be clear, correct, and unambiguous. Any typographical or grammatical errors should be corrected at revision, so please note any specific errors here.

Reviewer #1: No

Reviewer #2: No

**Answer:**

**We are appreciating your helpful advice. We are extensively checking and correcting the language or grammatical error according to reviewers comment and PLOS Global Public Health Journal standards.**

**5. Review Comments to the Author**

Please use the space provided to explain your answers to the questions above. You may also include additional comments for the author, including concerns about dual publication, research ethics, or publication ethics. (Please upload your review as an attachment if it exceeds 20,000 characters)

Reviewer #1: Before being accepted for publication, the manuscript needs to be extensively revised to strengthen its language. I have provided my suggested edits for areas needing attention (highlighted in yellow). The authors may consider them during revision.

Reviewer #2: I appreciate the opportunity to review this manuscript. I applaud the authors for this study and believe it is important to understand the factors affecting outcomes of burn injuries, especially in the resource limited settings. However, I have several points of feedback:

1. Regarding patients with mortality as an outcome, it’s important to exclude them from the ‘discharged with a complication’ category. More information on these cases should be provided for both groups. Additionally, clarification is needed on what was meant by a ‘scar of skin graft,’
**Answer: Great thanks for your view and thoughtfully seen .we are replaced by skin contracture because scaring is normal healing process**.
2. Clarification required on the assessment of burn injuries: The variables used appear to be quite broad, making it challenging to distinguish between actual complications and well-healed post-burn scars. In line 239, more information on how the researchers extracted details regarding specific complications should be provided. For instance, the variable "Did your upper extremity affected?" is mentioned, but it lacks specification regarding affected joints and range of motion. This omission would hinder the ability to determine whether a post-burn contracture or an amputation actually occurred.

**Answer: we are accepted and amend it**

Furthermore, categorizing "scarring at discharge" as a complication might be misleading, as this is a normal part of the healing process following a burn injury.
**Answer: Great thanks for your view and thoughtfully seen. We are replaces by skin contracture because scaring is normal healing process.**

3. The study appears limited in its consideration of comorbidities, focusing only on epilepsy. It’s essential to include other comorbidities like diabetes and heart disease, as they can significantly affect outcomes.
**Answer: we are accepted and amend it**

4. In the discussion section, it would be valuable for the authors to outline the burn treatment protocols followed at the hospitals studied. These protocols can have a substantial impact on burn outcomes.

5. The conclusion (line 292) mentions “a significantly higher level of outcome for burn injuries discharged with complications, leading to death and other worst outcomes.” The phrasing here is unclear. Please rephrase it for proper reporting.

**Answer: we are accepted and amend it**
6. Lastly, there is a need for extensive grammar editing throughout the manuscript.

6. PLOS authors have the option to publish the peer review history of their article ([what does this mean?](https://journals.plos.org/globalpublichealth/s/editorial-and-peer-review-process#loc-peer-review-history)). If published, this will include your full peer review and any attached files.

**Do you want your identity to be public for this peer review?** If you choose “no”, your identity will remain anonymous but your review may still be made public.

For information about this choice, including consent withdrawal, please see our [Privacy Policy](https://www.plos.org/privacy-policy).

Reviewer #1: **Yes:** Dehao Chen

Reviewer #2: No

**Answer: we are accepted and amend it**
