# Supplementary material for: Outcome of burn injury and its associated factors among burn patients attending public hospitals in North Showa Zone, Ethiopia: A cross-sectional study
Source: PLOS Glob Public Health. 2024 Sep 16;4(9):e0003682. doi: 10.1371/journal.pgph.0003682 (PMC11404801; doi:10.1371/journal.pgph.0003682)
Supplement: Attachment — Submitted filename: Response to reviewers.docx [file pgph.0003682.s007.docx]

**Letter of response**

**To all honorable reviewers and editors**

Dear Reviewers and Editors, We are very appreciative for your relevant comments, questions and recommendations. Following this, we have provided our responses point by point as presented below.

**Authors Responses**

1. If the authors have adequately addressed your comments raised in a previous round of review and you feel that this manuscript is now acceptable for publication, you may indicate that here to bypass the “Comments to the Author” section, enter your conflict of interest statement in the “Confidential to Editor” section, and submit your "Accept" recommendation.

Reviewer #1: (No Response)

Reviewer #2: (No Response)

**Answer for Reviewers**

We are appreciating for your view and thought fully review of our manuscript. Now we are intensively and deeply amend for the reviewers comment. As much possible we are kindly correct the grammatical error and address all comments.

2. Does this manuscript meet PLOS Global Public Health’s publication criteria? Is the manuscript technically sound, and do the data support the conclusions? The manuscript must describe methodologically and ethically rigorous research with conclusions that are appropriately drawn based on the data presented.

Reviewer #1: No

Reviewer #2: Partly

**Answer: we are accepted and amend it.**

3. Has the statistical analysis been performed appropriately and rigorously?

Reviewer #1: (No Response)

Reviewer #2: I don't know

**Answer: We are appreciating for your view and thought fully review of our manuscript. But our outcome variable is categorical and binary; we use binary logistic regression analysis methode. And also before analysis, we checked Goodness of fitness was assessed by Hosmer-Lemeshow test & interpret in rigorously**

4. Have the authors made all data underlying the findings in their manuscript fully available (please refer to the Data Availability Statement at the start of the manuscript PDF file)?

The PLOS Data policy requires authors to make all data underlying the findings described in their manuscript fully available without restriction, with rare exception. The data should be provided as part of the manuscript or its supporting information, or deposited to a public repository. For example, in addition to summary statistics, the data points behind means, medians and variance measures should be available. If there are restrictions on publicly sharing data—e.g. participant privacy or use of data from a third party—those must be specified.

Reviewer #1: (No Response)

Reviewer #2: Yes

**Answer:**

**We are appreciating your helpful advice. We are extensively checking and correcting the language or grammatical error according to reviewers comment and PLOS Global Public Health Journal standards.**

**5**. Is the manuscript presented in an intelligible fashion and written in Standard English?

PLOS Global Public Health does not copyedit accepted manuscripts, so the language in submitted articles must be clear, correct, and unambiguous. Any typographical or grammatical errors should be corrected at revision, so please note any specific errors here.

Reviewer #1: No

Reviewer #2: No

**Answer: we are accepted and amend it**.

**6.** **Review Comments to the Author**

Please use the space provided to explain your answers to the questions above. You may also include additional comments for the author, including concerns about dual publication, research ethics, or publication ethics. (Please upload your review as an attachment if it exceeds 20,000 characters)

**Reviewer #1:** To evaluate this revision, a spot check was conducted for the sections before Result to assess if reviewers' comments/suggestions from the last round of review have been incorporated. While a few suggested edits/comments have been taken into account, the revision does not reflect other suggestions - the authors should explain why those comments were not taken into account. Examples of the areas without considering reviewer's suggestions were highlighted in the attachment. I highly recommend the authors consider the suggested edits that have not been adopted to improve the writing.

Further, seemingly a reviewer's comment was directly pasted to the manuscript (lines 80-82). Copyediting errors like this should be prevented.

**Reviewer #2:** Thank you for inviting me to review this manuscript. I commend the authors for the extensive work done to develop this interesting research.

However, I recommend that this manuscript undergo extensive English revision, preferably by an expert in scientific writing, to ensure better presentation before publication.

**Answer: we are accepted and amend it**

**7.** PLOS authors have the option to publish the peer review history of their article (what does this mean?). If published, this will include your full peer review and any attached files.

**Do you want your identity to be public for this peer review**? If you choose “no”, your identity will remain anonymous but your review may still be made public.

For information about this choice, including consent withdrawal, please see our Privacy Policy.

Reviewer #1: No

Reviewer #2: No

**Answer: we are accepted and amend it**
