# Supplementary material for: Outcome of burn injury and its associated factors among burn patients attending public hospitals in North Showa Zone, Ethiopia: A cross-sectional study
Source: PLOS Glob Public Health. 2024 Sep 16;4(9):e0003682. doi: 10.1371/journal.pgph.0003682 (PMC11404801; doi:10.1371/journal.pgph.0003682)
Supplement: Attachment — Submitted filename: Response to reviewers.docx [file pgph.0003682.s008.docx]

**Letter of response**

**To all honorable reviewers and editors**

Dear Reviewers and Editors, We are very appreciative for your pertinent comments, questions and recommendations. Following this, we have provided our responses point by point as presented below.

**Authors Responses**

1. If the authors have adequately addressed your comments raised in a previous round of review and you feel that this manuscript is now acceptable for publication, you may indicate that here to bypass the “Comments to the Author” section, enter your conflict of interest statement in the “Confidential to Editor” section, and submit your "Accept" recommendation.

Reviewer #1: (No Response)

Reviewer #2: (No Response)

**Answer for Reviewers**

**We addressed the reviewers comment in depth and carefully edit the grammar, punctuation mark, technical terms, finding interpretation, all the reviewers comment that color on revised manuscript for track change. And also we have been written in Standard English and comprehensive written fashion based on PLOS Global Public Health’s publication criteria with reference of language expert and consultant. If this manuscript is accepted for publication, researchers, decision makers, and health care providers add new knowledge for reduction and effective management of burn injuries.**

2. Does this manuscript meet PLOS Global Public Health’s publication criteria? Is the manuscript technically sound, and do the data support the conclusions? The manuscript must describe methodologically and ethically rigorous research with conclusions that are appropriately drawn based on the data presented.

Reviewer #1: No

Reviewer #2: Partly

**Answer for reviewers**

**The finding of thise manuscript were drawn based on the data that statistically significant to conclude the result. Our study design is cross-sectional, so that we described the magnitude of burn outcome and associated factors that independently change the dependent variables at 95% CI. The ethical consideration of the research also fulfilled the research ethics because we were incorporated the basic principles of research ethics like confidentiality of the study participants, informed consent before data collection and etc.**

3. Has the statistical analysis been performed appropriately and rigorously?

Reviewer #1: (No Response)

Reviewer #2: I don't know

**Answer for reviewers**

**Our statistical analysis model was binary logistic regression because of the outcome variable is dichotomous (yes/no). The stastical analysis has been performed strictly and appropriately that means all assumption of the binary logistic regression were fulfilled and encompassed. Hence, the statistical analysis is accurate and reliable that described the interesting finding manuscript.**

4. Have the authors made all data underlying the findings in their manuscript fully available (please refer to the Data Availability Statement at the start of the manuscript PDF file)?

The PLOS Data policy requires authors to make all data underlying the findings described in their manuscript fully available without restriction, with rare exception. The data should be provided as part of the manuscript or its supporting information, or deposited to a public repository. For example, in addition to summary statistics, the data points behind means, medians and variance measures should be available. If there are restrictions on publicly sharing data—e.g. participant privacy or use of data from a third party—those must be specified.

Reviewer #1: (No Response)

Reviewer #2: Yes

**Answer for reviewer- 1**

**All the available data finding are fully available in our manuscript. And also we are described in the data availability statement.**

**Answer for reviewer 2**

**N/A**

**5**. Is the manuscript presented in an intelligible fashion and written in Standard English?

PLOS Global Public Health does not copyedit accepted manuscripts, so the language in submitted articles must be clear, correct, and unambiguous. Any typographical or grammatical errors should be corrected at revision, so please note any specific errors here.

Reviewer #1: No

Reviewer #2: No

**Answer for reviewers**

**Our manuscript is original that means thise research finding is done only ours and not copy edited that checked online plagiarism checker. The writing language was written by using Standard English, clear, valuable, independent, not used jargon words, and comprehensive written fashion based on PLOS Global Public Health’s publication criteria with reference of language expert and consultant.**

**6.** **Review Comments to the Author**

Please use the space provided to explain your answers to the questions above. You may also include additional comments for the author, including concerns about dual publication, research ethics, or publication ethics. (Please upload your review as an attachment if it exceeds 20,000 characters)

**Reviewer #1:** To evaluate this revision, a spot check was conducted for the sections before Result to assess if reviewers' comments/suggestions from the last round of review have been incorporated. While a few suggested edits/comments have been taken into account, the revision does not reflect other suggestions - the authors should explain why those comments were not taken into account. Examples of the areas without considering reviewer's suggestions were highlighted in the attachment. I highly recommend the authors consider the suggested edits that have not been adopted to improve the writing.

Further, seemingly a reviewer's comment was directly pasted to the manuscript (lines 80-82). Copyediting errors like this should be prevented.

**Reviewer #2:** Thank you for inviting me to review this manuscript. I commend the authors for the extensive work done to develop this interesting research.

However, I recommend that this manuscript undergo extensive English revision, preferably by an expert in scientific writing, to ensure better presentation before publication.

**7.** PLOS authors have the option to publish the peer review history of their article (what does this mean?). If published, this will include your full peer review and any attached files.

**Do you want your identity to be public for this peer review**? If you choose “no”, your identity will remain anonymous but your review may still be made public.

For information about this choice, including consent withdrawal, please see our Privacy Policy.

Reviewer #1: No

Reviewer #2: No

**Answer**

**We accept and do it**

**We thanks to much for your quick response!!!!!!!**
