# Supplementary material for: Outcome of burn injury and its associated factors among burn patients attending public hospitals in North Showa Zone, Ethiopia: A cross-sectional study
Source: PLOS Glob Public Health. 2024 Sep 16;4(9):e0003682. doi: 10.1371/journal.pgph.0003682 (PMC11404801; doi:10.1371/journal.pgph.0003682)
Supplement: Attachment — Submitted filename: Response to reviewers.docx [file pgph.0003682.s010.docx]

**Letter of response**

**To all honorable reviewers and editors**

Dear Reviewers and Editors, We are very appreciative for your pertinent comments, questions and recommendations. Following this, we have provided our responses point by point as presented below.

**Authors Responses**

Background: Burn injury is a major contributor to morbidity and mortality in Sub-Saharan Africa, including Ethiopia. require regular interventions???.There is limited evidence on outcome burn injury status??? in Ethiopia including the study area (specify?).

**.**

**Objective**: To assess the outcome of burn injury and its associated factors (unclear) among burn patients attending ? a public Hospital (which one?) in North Showa zone, Ethiopia.2023 (unclear)

Method: An institution-based, cross-sectional study was conducted among 420 burn patients in a public hHospital in North Showa zone, from April to May, 2023. Systematic random sampling was used to select patients’ cards???. Structured checklists were used to extract data from burn patients’ medical records. Data were entered using Epi-data EpiData version 4.6 and analyzed using SPSS version 25 variables with a p- value??? unclear of ≤ 0.25 in the bivariable logistic regression were included in the multivariable logistic regression. A p- value of ≤ 0.05 within 95% confidence interval in the multivariable logistic regression was used to declare significant association between the dependent variable and the independent variables. Adjusted odds ratio was used to show the strength of association between the dependent and independent variables. Result: In this study, 40.9% (95% CI: 36.5– 45.6) of patients were discharged with complications.  Patients who had pre-hospital intervention was nearly four times (AOR= 3.8, 95% CI, 1.11-13.25) and Patients burn with scalds was four (AOR=4.3, 95% CI, 1.52-12.32) times more (break into 2 sentences please or improve the conjunction) likely to develop discharged with complication. <Unclear Patients who had provided fluid and electrolyte were 76% and the occurrence of total body surface area burn  <Unclear etc.

**Answer:**

**The first line indicates, the burden or severity and impact of burn injury in Ethiopian population, So that it requires urgent intervention. The study area is North showa zone, Amhara regional state, Ethiopia**

**The objectives of this study were the prevalence /magnitude and associated factors /risk factors of burn injury in public hospitals of North showa, Zone, Ahmara region, Ethiopia. There are 11 hospitals in North showa, zone.**

**The other comments are corrected and amended.**

**We thanks to much for your quick response!!!!!!!**
