# Supplementary material for: Outcome of burn injury and its associated factors among burn patients attending public hospitals in North Showa Zone, Ethiopia: A cross-sectional study
Source: PLOS Glob Public Health. 2024 Sep 16;4(9):e0003682. doi: 10.1371/journal.pgph.0003682 (PMC11404801; doi:10.1371/journal.pgph.0003682)
Supplement: Attachment — Submitted filename: Response to reviewers.docx [file pgph.0003682.s013.docx]

**Letter of response**

**To all honorable reviewers and editors**

Dear Reviewers and Editors, We are very appreciative for your pertinent comments, questions and recommendations. Following this, we have provided our responses point by point as presented below.

**Authors Responses**

1. If the authors have adequately addressed your comments raised in a previous round of review and you feel that this manuscript is now acceptable for publication, you may indicate that here to bypass the “Comments to the Author” section, enter your conflict of interest statement in the “Confidential to Editor” section, and submit your "Accept" recommendation.

Reviewer #1: (No Response)

Reviewer #2: All comments have been addressed

**Answer**

**We are carefully and enthusiastically amended the comments arise by reviewers.**

2. Does this manuscript meet PLOS Global Public Health’s [publication criteria](https://journals.plos.org/globalpublichealth/s/criteria-for-publication)? Is the manuscript technically sound, and do the data support the conclusions? The manuscript must describe methodologically and ethically rigorous research with conclusions that are appropriately drawn based on the data presented.

Reviewer #1: Partly

Reviewer #2: Yes

**Answer**

**Our manuscript was methodologically and ethically rigorous and the conclusion was based the presented data. Our manuscript also very important for public health and the data supported its conclusion. Our manuscript also meets PLOS Global Public Health criteria by amended by reviewer comments.**

3. Has the statistical analysis been performed appropriately and rigorously?

Reviewer #1: (No Response)

Reviewer #2: I don't know

**Answer**

**The statistical analysis was performed based on the outcome variable that means our outcome variable was dichotomous so that we used binary logistic regression method.**

4. Have the authors made all data underlying the findings in their manuscript fully available (please refer to the Data Availability Statement at the start of the manuscript PDF file)?
The [PLOS Data policy](https://journals.plos.org/globalpublichealth/s/data-availability) requires authors to make all data underlying the findings described in their manuscript fully available without restriction, with rare exception. The data should be provided as part of the manuscript or its supporting information, or deposited to a public repository. For example, in addition to summary statistics, the data points behind means, medians and variance measures should be available. If there are restrictions on publicly sharing data e.g. participant privacy or use of data from a third party those must be specified.

Reviewer #1: (No Response)

Reviewer #2: Yes

**Answer**

**We accepted the comments and amend it carefully.**

5**.** Is the manuscript presented in an intelligible fashion and written in Standard English?
PLOS Global Public Health does not copyedit accepted manuscripts, so the language in submitted articles must be clear, correct, and unambiguous. Any typographical or grammatical errors should be corrected at revision, so please note any specific errors here.

Reviewer #1: (No Response)

Reviewer #2: Yes

**Answer**

**Our manuscript was written in Standard English and intellectual fashion based our reviewers comment, and not copy edited because the research article is originally prepared by ours.**

6. Review Comments to the Author
Please use the space provided to explain your answers to the questions above. You may also include additional comments for the author, including concerns about dual publication, research ethics, or publication ethics. (Please upload your review as an attachment if it exceeds 20,000 characters)

**Reviewer #1**: I appreciate the authors' consideration of my comments from the prior reviews. The manuscript looks much better now. However, before it being accepted for publication. There are still things that can be improved, which are listed as comments in the upload.
I'd appreciate if the authors can use track changes for the next (but hopefully final) round of review. Also, I find it challenging to open the uploaded figures. Can they be uploaded in a more common format (e.g., tiff, jpeg). Lastly, it'd be helpful to replace the long web links of some citations with DOIs of the cited articles.

**Answer for reviewer 1**

**We are amend the above comments includes DOIs of the cited articles, all comments in the attached file like exclusion criteria, variables, abstract methods.**

**Reviewer #2**: I congratulate the authors and the editorial team for their tireless efforts to improve this great work. I suggest some minor edits in the abstract which still seem unclear, before publication.
-Line 35-37: "Patients who received total body surface area were 20% less likely to develop discharge with complications than those who had not burned"
Line 40: “Therefore, stakeholder would more emphasis in health education on pre-hospital intervention…….”
Congratulations, and I recommend this work to be published.

**Answer for reviewer 2**

**We are amending the two comments enthusiastically**.

7. PLOS authors have the option to publish the peer review history of their article ([what does this mean?](https://journals.plos.org/globalpublichealth/s/editorial-and-peer-review-process#loc-peer-review-history)). If published, this will include your full peer review and any attached files.
**Do you want your identity to be public for this peer review?** If you choose “no”, your identity will remain anonymous but your review may still be made public.
For information about this choice, including consent withdrawal, please see our [Privacy Policy](https://www.plos.org/privacy-policy).

Reviewer #1: No

Reviewer #2: No

**“We thanks to much for your quick response!!!!!!!**
