# Supplementary material for: Outcome of burn injury and its associated factors among burn patients attending public hospitals in North Showa Zone, Ethiopia: A cross-sectional study
Source: PLOS Glob Public Health. 2024 Sep 16;4(9):e0003682. doi: 10.1371/journal.pgph.0003682 (PMC11404801; doi:10.1371/journal.pgph.0003682)
Supplement: Attachment — Submitted filename: Response to reviewers.docx [file pgph.0003682.s014.docx]

**Letter of response**

**To all honorable reviewers and editors**

Dear Reviewers and Editors, We are very appreciative for your pertinent comments, questions and recommendations. Following this, we have provided our responses point by point as presented below.

**Authors Responses**

1. Line 36-37: Please change the sentence that says, "Patients who received total body surface area were 20% less likely to develop discharge with burn complications than those who had not burned" to '**'Patients with TBSA less than 20% were 66% less likely to be discharged with complications compared to patients with TBSA greater than 20%**"
2. Line 212: Steam is still a form of a scald burn. Please sub-categorize scald into ''hot liquids" and "steam" in order to keep the results and figures unaffected.
3. Line 265: Replace 21% with 20%

**Answer**

**We are accepted your valuable comments and amend in point by point.**

**“We thanks to much for your quick response!!!!!!!**
